# Lexical word processing is unaffected by rapid invisible frequency tagging in reading: Evidence from eye movements

**DOI:** 10.3758/s13423-026-02948-x

**Published:** 2026-06-30

**Authors:** Veronika Prigorkina, Heather Sheridan, Steven Frisson, Yali Pan

**Affiliations:** 1 Department of Experimental and Applied Psychology, Vrije Universiteit Amsterdam, Amsterdam, The Netherlands; 2 Department of Psychology, University at Albany, Albany, NY USA; 3 Centre for Human Brain Health, School of Psychology, University of Birmingham, Birmingham, UK

**Keywords:** Rapid invisible frequency tagging (RIFT), Reading, Parafoveal processing, Word processing, Word frequency effect, Eye-tracking, Survival analysis

## Abstract

The aim of this study was to test whether rapid invisible frequency tagging (RIFT)—a promising tool to directly measure covert attention allocated to parafoveal words—affects lexical processing during natural reading. RIFT involves flickering the background of the target word(s) at an imperceptible frequency (≥60 Hz), but imperceptibility does not necessarily equate to the absence of a cognitive effect. The current study examined the potential impact of the RIFT paradigm on eye movements. Forty-eight participants read 474 high-frequency and low-frequency target words that were embedded in one-line sentences and displayed randomly in three condition blocks: no tagging, invisible tagging at 60 Hz (i.e., the RIFT) and visible tagging at 30 Hz. Linear mixed-effect modelling and divergence point analysis revealed a robust frequency effect, with high-frequency words processed faster than low-frequency words, but, crucially, no significant differences across the three conditions in terms of its magnitude or onset latency, except for a miniscule effect on saccade amplitude (0.06˚). Overall fixation durations (irrespective of lexical frequency) did not differ between the no-tagging and RIFT conditions, but were significantly longer in the visible tagging condition, suggesting that deliberately drawing attention to the tagging area actually slowed down reading. Furthermore, a postexperiment questionnaire indicated relatively low RIFT awareness. Altogether, preserved lexical frequency effects of comparable magnitude, similar divergence points and overall fixation and saccade patterns, suggest that RIFT is a valid tool for measuring attention during reading that is unlikely to interfere with word processing and highlight its potential for application in ecologically valid settings involving eye movements.

**Supplementary Information:**

The online version contains supplementary material available at https://doi.org/10.3758/s13423-026-02948-x.

## Introduction

Reading is a uniquely human ability that relies on a wide range of cognitive processes. Among these, attention plays a central role, regulating the flow of textual information and acting as a catalyst for reading fluency (Commodari & Guarnera, 2005; Valdois et al., 2019). A long-standing debate in the literature concerns how attention is distributed during reading. Some accounts suggest that attention shifts from one word to the next in a serial manner (Reichle et al., 1998; Salvucci, 2001), while other frameworks claim it is allocated to several words at a time (Engbert et al., 2005; Jensen et al., 2021; Reilly & Radach, 2006; Snell, 2024; Snell et al., 2018). The ever-growing scientific interest in this research field increasingly motivates the use of novel techniques and approaches.

Among these, rapid invisible frequency tagging (RIFT) stands out as a particularly promising approach, distinguished by its ability to capture the dynamic allocation of attention during natural reading and the inherent ecological validity of its procedure. RIFT is a high-frequency (≥60 Hz) tagging technique that involves periodic changes in luminance of the visual stimuli (Duecker et al., 2021, 2025; Ferrante et al., 2023; Zhigalov et al., 2019). In the scenario of natural reading, RIFT modulates the luminance of a part of the screen underlying the region(s) of interest. Since such tagging is around the visual fusion threshold, the flickering patch is perceived as invisible to the readers but evokes a characteristic neural response in the visual cortex, manifested as alignment in frequency of the visual cortex activity and the luminance signal. Such a paradigm allows calculating the extent of attention allocated to the flickering area even before a saccade to that region is made (i.e., the covert attention during parafoveal processing; Pan et al., 2021, 2023, 2025). This suggests RIFT is an effective tool for studying attention during natural reading, which has already contributed valuable information to the debate about serial vs. parallel word processing (e.g., Jensen et al., 2021; Pan et al., 2021, 2023, 2025).

Recent RIFT studies have shown that readers gain access to lexical and semantic information about the upcoming word as early as 90–100 ms after the first fixation on the preceding word (Pan et al., 2021, 2023, 2025). Since other techniques, such as fixation-related-potentials (Antúnez, Milligan, et al., 2022b; Degno et al., 2019; Li et al., 2024; López-Peréz et al., 2016; Milligan, Antúnez et al., 2023a; Milligan, Nestor et al., 2013b; Niefind & Dimigen, 2016; Payne et al., 2019; Schotter et al., 2023), have generally failed to capture such early parafoveal-on-foveal effects, it is fair to ask whether there are properties of the RIFT paradigm that might have induced or exacerbated these effects. Concretely, even though participants are generally not consciously aware of the flickering manipulation (Pan et al., 2021; Spaak et al., 2024), the presence of the flicker might still attract (additional) attention to the flickering area, resulting in enhanced parafoveal processing. This would allow for an alternative explanation of the lexico-semantic parafoveal-on-foveal effects in the tagging-induced signal—namely, as a byproduct of the flickering, as long as such a manipulation can affect lexical word processing.

While it has been shown that allocation of visuo-spatial attention is modulated by foveal and parafoveal processing load (Antúnez, López-Pérez, et al., 2022a; Henderson et al., 1990; Pan et al., 2025; Payne et al., 2016; Schroyens et al., 1999; Veldre & Andrews, 2018; White et al., 2005), there is no conclusive evidence suggesting that changes in attention distribution could *alter* lexical word recognition (Ekstrand et al., 2016; McCann et al., 1992). At the same time, it is not inconceivable that stimulating the reader to allocate more attention to the upcoming parafoveal regions would result in more subtle changes to the *time course* or *depth* of parafoveal and foveal lexical word processing. For instance, by attracting more covert attention to the upcoming parafoveal word, RIFT could result in more efficient consequent foveal processing of the tagged regions and/or exacerbated parafoveal-on-foveal effects from these regions, possibly at the cost of the preceding foveal word’s processing. For these reasons, we believe it is important to explore the influence of tagging both on global reading patterns and lexical word processing.

At the same time, if RIFT indeed attracts more attention towards the tagged area, this effect should be even more pronounced in consciously perceptible flickering at lower frequencies, since it is more easily identifiable and may automatically draw attention. This is supported by studies from the domain of spatial attention, suggesting that only perceptible (20–48 Hz) but not subliminal (60–96 Hz) flickering, both brief and long, can serve as a robust exogenous attention cue and automatically draw attention to the tagged area (Alais et al., 2016; Stolte & Ansorge, 2021). Similarly, studies with chromatic flicker that use colour rather than luminance also show that higher-frequency imperceptible flickering is less likely to attract exogenous attention (Lu et al., 2012; Zhang et al., 2021).

While there is no direct evidence for the influence of RIFT on reading, related studies have explored the effect of whole-text flickering induced by LED lighting, Lexilens glasses, full-screen tagging, and monitor refresh rate. Some studies suggest that high-frequency flickering disrupts oculomotor control in typical readers, leading to greater variability in saccade landing positions, saccade length, and refixation probability (50 Hz and 100 Hz; Kennedy & Murray, 1991; Wilkins, 1986), or slows down reading of visually demanding texts (60 Hz; Laycox et al., 2024). In contrast, Le Floch & Ropars (2023) found no adverse effect of high-frequency flickering in typical readers, but reported enhanced reading fluency in readers with dyslexia, an effect that was not replicated in another study (Lubineau et al., 2023; see also Kodochian, 2024). At lower frequencies (≤30 Hz), visible flickering has been shown to interfere with word recognition in typical readers (Lubineau et al., 2023), but to improve letter identification and word reading for a single reader with dyslexia (McCloskey & Rapp, 2000). Taken together, the evidence for flicker effects on reading behaviour remains inconsistent, highlighting the need for further investigation.

### The present study

Here we address the following two research questions:

1. Does RIFT at 60 Hz impact lexical word processing during natural reading and
2. Does the visibility of the flicker play a role in this?

While there are reasons to suspect that RIFT facilitates lexical word processing, previous studies suggest that both visible and invisible tagging may disrupt the reading process (Kennedy & Murray, 1991; Laycox et al., 2024; Lubineau et al., 2023; Wilkins, 1986). Both possibilities would be similarly unfortunate for RIFT as a method to study covert attention in reading. At the same time, any effect of RIFT is expected to be exacerbated in consciously perceptible flickering. This exploration is, therefore, crucial for both future studies employing RIFT and further advancing the theory of eye movement control during reading.

**Fig. 1:**
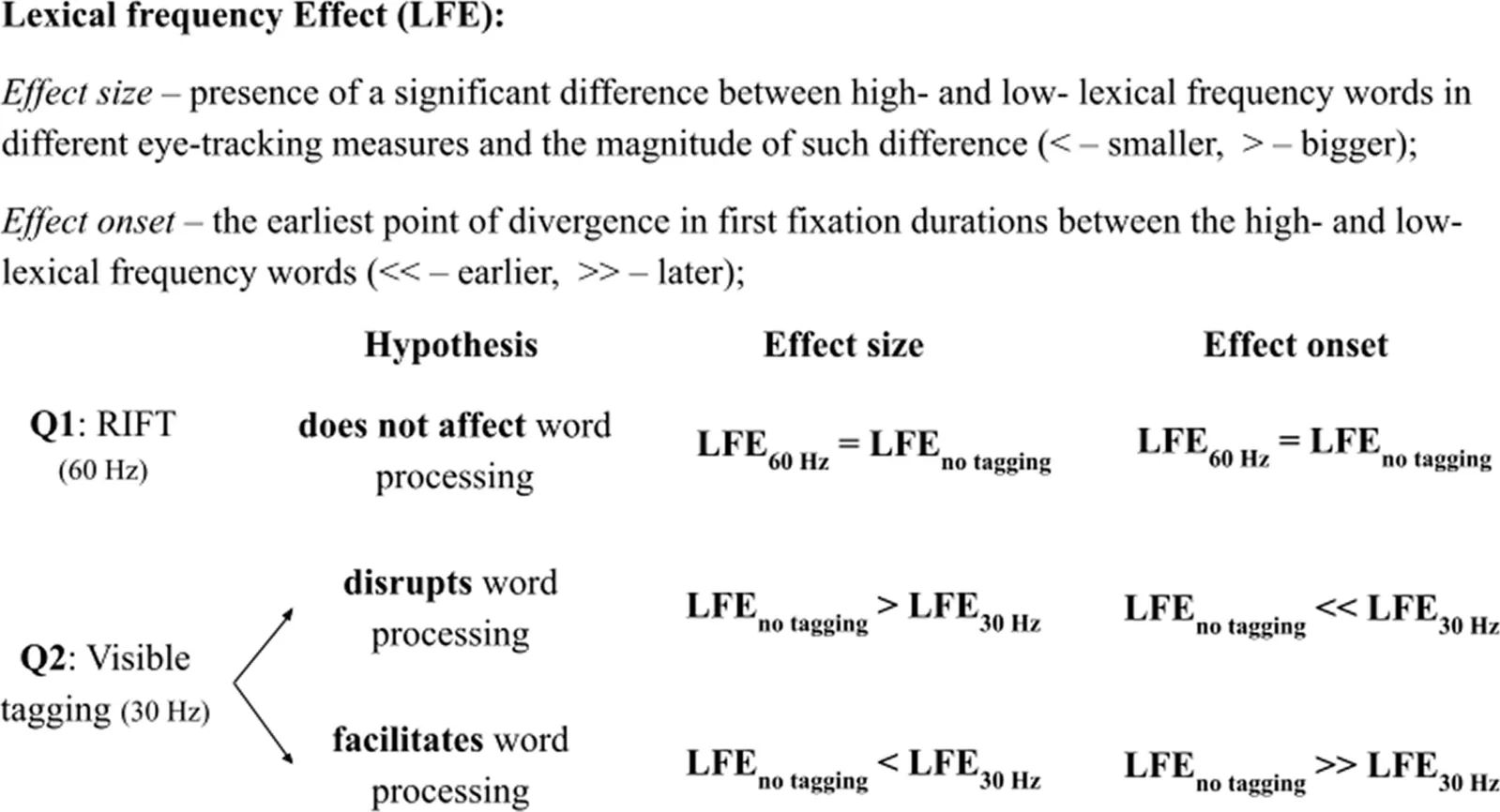
Hypotheses diagram

For that purpose, we conducted an eye-tracking experiment in which participants read individual sentences silently at their own pace. We manipulated the lexical frequency of a target word in each sentence (low vs. high) and the presentation mode of these sentences (no tagging, visible tagging, and RIFT). By observing the differences in processing of more and less frequent words in different tagging conditions, we investigated possible deviations in lexical word processing when exposed to more or less visible flickering. The *lexical frequency effect*—that is, the processing advantage of high-frequency (HF) words over low-frequency (LF) words, was used as a proxy of lexical word processing. The lexical frequency effect has consistently been found in eye-tracking studies, where HF words are fixated for a shorter time and are skipped more frequently (Inhoff & Rayner, 1986; Rayner & Duffy, 1986; Rayner et al., 1996; Schilling & Chumbley, 1998; Staub et al., 2010; White, 2008). Since the lexical frequency effect serves as a robust marker of early lexical word processing during reading, its size and timing was compared across the tagging conditions using linear mixed-effects (LME) analysis and divergent point analysis (DPA; see the Data Analysis section for details). For RIFT, we expected to see no deviations in lexical frequency effect size or onset, reflecting lexical word processing comparable to natural reading. If the visible 30-Hz tagging facilitates word processing during reading, we anticipated a larger lexical frequency effect size and an earlier lexical frequency effect onset than in the no-tagging condition. Alternatively, if visible tagging disrupts lexical processing, we expected to see a smaller lexical frequency effect size and a later lexical frequency effect onset (see Fig. 1).

## Methods

### Participants

Forty-eight native British English speakers (age range 18–44 years, *M* = 20.24, *SD* = 2.44) participated in the experiment.^1^ All of the participants had normal or corrected-to-normal vision and reported no history of reading difficulties (e.g., dyslexia) or neurodevelopmental disorders (e.g., ADHD, epilepsy). All the participants provided written informed consent and received £15 per hour for their participation.

Sample size was preregistered^2^ and determined based on prior reading studies that used the divergent point analysis (DPA) and on linear mixed-effects (LME) analysis guidelines for simpler designs. For DPA, a power simulation study showed that a sample of around 50 participants introduces little-to-no bias^3^ in the divergence point estimates, given at least 60 observations per condition (Reingold & Sheridan, 2014). In the current study a total of 48 participants read 97 trials per condition, for a maximum total of 4,656 observations per condition, which is roughly in line with the requirement of at least 1,600 observations per condition and a minimum of 50 participants in a repeated-measures design (Brysbaert, 2019; Brysbaert & Stevens, 2018). Therefore, the sample size was deemed sufficient for both the DPA and LME analyses.

### Design

The current study employed a repeated-measures block design. Two factors were manipulated: the lexical frequency of the target word (high vs. low) and the tagging conditions (no-tagging vs. RIFT modulation at 60 Hz vs. visible tagging at 30 Hz). The three tagging conditions were presented in three separate blocks, and the order of these blocks was randomized across participants. Within each of these blocks, all sentences were presented in the same tagging condition. Each sentence was presented to each participant only once, either with a low or with a high lexical frequency target word and only in one of the three tagging blocks, counterbalanced across participants. Within each block, stimuli were randomized so that no more than three consecutive trials were from the same lexical frequency condition.

### Material

A set of 474 sentences from three different studies was used (Degno et al., 2019; Pan et al., 2021; Schotter & Leinenger, 2016). Most sentences had a single target word per sentence (see Example 1, below), except a subset of stimuli from Degno and colleagues (2019), where each sentence contained two target words (see Example 2 below), both of either high frequency (HF) or low lexical frequency (LF). Overall, these sets of stimuli comprised 582 target words distributed over 474 sentences, with HF words having a significantly higher log frequency than LF words (HF: *M* = 233.23, *SD* = 316.00; LF: *M* = 8.69, *SD* = 4.98). Word length was identical for high-lexical and low-lexical frequency target words in each sentence of each stimuli dataset (*M* = 5.80, *SD* = 0.80 in Pan et al., 2021; *M* = 5.59, *SD* = 0.49 in Schotter & Leinenger, 2016; *M* = 5.88, *SD* = 0.83 in Degno et al., 2019). All sentences were plausible and relatively unpredictable (see Supplementary Materials for more details), occupied a single line of text, and were evenly distributed across the six experimental conditions (2 lexical frequency conditions × 3 tagging conditions; 79 sentences and 97 target words per condition). Examples:

1. It was obvious that the beautiful *music/waltz* captured her attention. (from Pan et al., 2021)
2. I bought a black *guitar/kettle* last summer, and the pricey *flight/violin* that Sam wanted. (from Degno et al., 2019)

### Procedure

The experimental setup was identical to previous MEG RIFT experiments (Pan et al., 2021, 2023, 2025) to ensure maximal replication of the experimental environment. The reading task was conducted in the MEG magnetically shielded room with dim lighting. Participants were comfortably seated under the MEG scanner. Their eye movements from the right eye were recorded throughout the whole session at a sampling rate of 1000 Hz using an EyeLink 1000 Plus system (SR Research Ltd, Canada), situated on a table in front of the participants. To minimize false saccade reports and reduce the occurrence of micro-saccades, we used the following parameters for the fixation events parsing that is suggested by the EyeLink manual for a reading study: a motion threshold of 0.1°, a velocity threshold of 30°/s, and an acceleration threshold of 8,000°/s.^2^ In addition, MEG data was collected for 27 participants but not analysed for the current study (see Pan et al., 2023, for details of the MEG recording setup).^4^

Stimuli were projected from the stimulus computer screen (120-Hz refresh rate; 1,920 × 1,080 pixels resolution) to the MEG-compatible projection screen, positioned at a distance of 100 cm from the participant’s eyes, using a PROPixx DLP LED projector (VPixx Technologies Inc., Canada) with a refresh rate of 1440 Hz. The sentences were presented in black, equally spaced Courier New font (18 pt., bold), on the middle grey (RGB [128 128 128]) screen background. Each character and the interword spaces occupied 0.5 visual degrees horizontally. A rectangular frequency-tagging patch was added underneath the target word. Its width was equal to the width of the target word plus the spaces on both sides, ranging from 2° to 4° visual angle. The patch was either constantly grey (in the no-tagging block) or it was flickered by altering its luminance from black to white at a sine wave of 60 Hz (RIFT block) or 30 Hz (visible tagging block).^5^

**Fig. 2:**
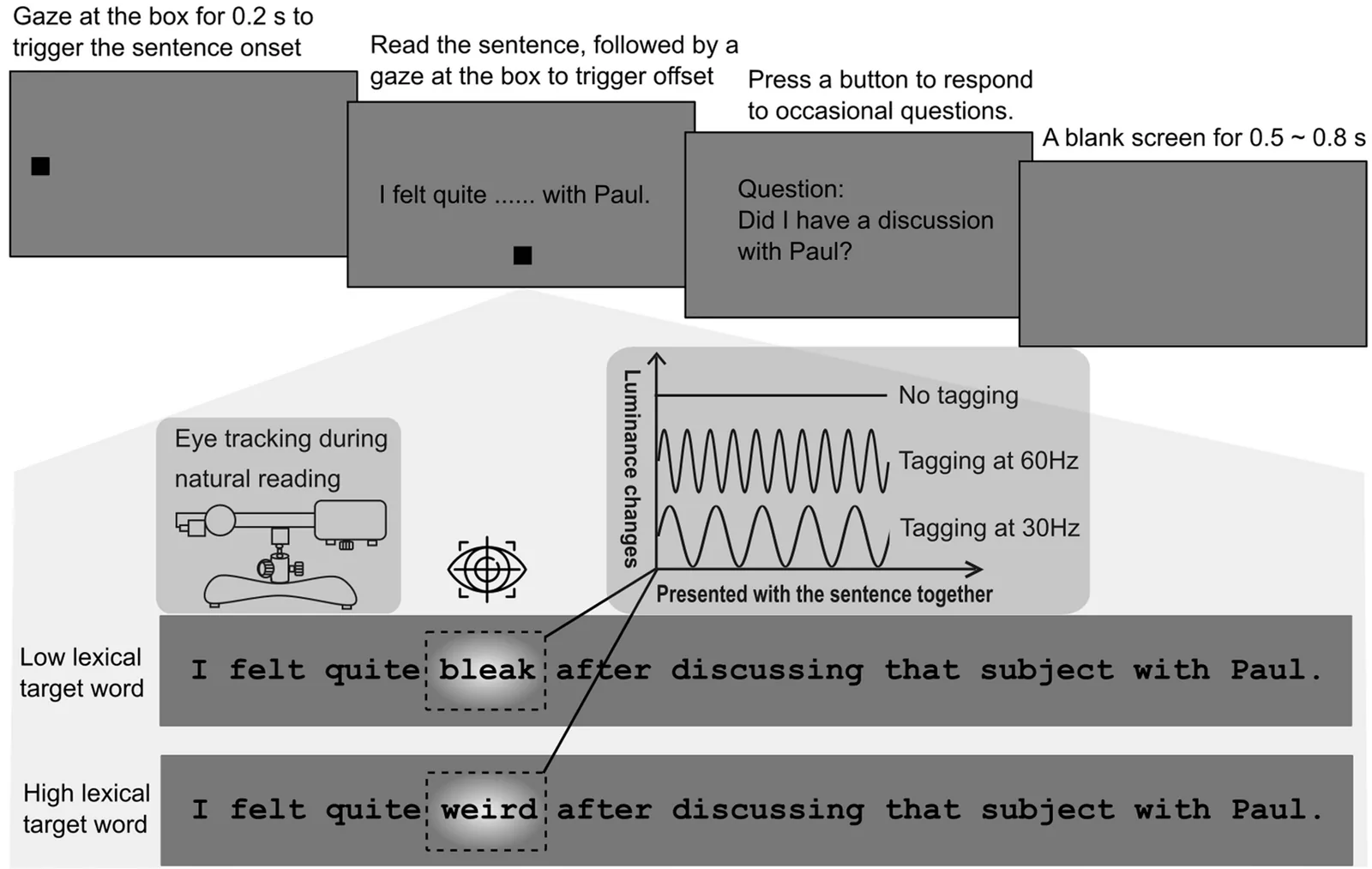
RIFT Eye-tracking experimental paradigm

The study consisted of three separate blocks corresponding to the three tagging conditions, with a 30-s break between each block. A third of the sentences was presented in the no-tagging condition, another third in the RIFT condition, and the final third in the visible tagging condition. The sequence of the tagging blocks was counterbalanced so that each participant completed the blocks in one of the six possible orders. Prior to commencing each block, we performed a 5-point calibration and validation procedure, ensuring a tolerated gaze-position error of no more than 1 visual degree (*M* = 0.37, *SD* = 0.13) along both the vertical and horizontal axes. Furthermore, a drift-correction check was performed every six trials to maintain this precision. As depicted in Fig. 2, at the beginning of each trial, a square with a diameter of 1° visual angle was presented, vertically centred and horizontally positioned 1.3° from the screen’s left edge. The position of the square aligned with the starting position of the first character of each sentence. When a fixation of minimum 200 ms was detected within the square’s boundary, the square disappeared and the sentence was automatically displayed. Participants were instructed to silently read the sentence at their own pace and then to gaze at a square located 2° below the sentence for 100 ms, which served to trigger the offset of the sentence. One-fifth of the sentences were followed by a comprehension question, where participants had to agree or disagree with a statement concerning the content of the sentence by using a button box. Accuracy was high (94%). The trial was concluded with a blank grey screen displayed for 0.5–0.8 s (randomly selected from a uniform distribution). The experiment lasted approximately 1.5 hours. All scripts were programmed in MATLAB R2020a (The MathWorks, Natick, MA, USA), using Psychophysics Toolbox-3 and custom-made scripts.

At the very end of the experiment, 44 participants were also asked to fill out a short questionnaire (see Supplementary Materials). Firstly, they were asked whether they thought the luminance of some words on the screen was changing during the experiment. Secondly, if participants responded to the first question positively, they were asked to indicate all the experimental blocks (first, second, and\or third, in their order of presentation) in which they saw flickering. Finally, for each block where they though there was flickering, participants were asked to indicate on a 6-point Likert scale how frequently they though it occurred throughout the trials. Response 0 corresponded to 0% of the trials and response 5 corresponded to 100% of the trials. If participants skipped this question, it was regarded as equal to response 0. The order of the blocks in the experiment for specific participants was later linked with corresponding tagging conditions, allowing to estimate the perceptibility of flickering in different tagging conditions across participants.

### Data analysis

To capture possible influences of flickering on both the size and the onset of the lexical frequency effect, we employed the LME analysis, the DPA, and the Bayesian*-*LME analysis. We decided to complement the classic frequentist methods with probabilistic (i.e., Bayesian) and nonparametric distributional analyses (i.e., the DPA) to provide a broader picture of the effects of frequency tagging on reading and to have greater assurance in case of null effects. For the RIFT sensitivity questionnaire, we performed paired two-sided *t* tests to check whether flickering is indeed more noticeable in the 30-Hz tagging than in the 60-Hz tagging condition.

#### Linear mixed-effect (LME) analysis

LME analyses (not preregistered) were performed to compare the size of the lexical frequency effect for different eye-movement measures. Since the objective of the current study is to investigate the validity of RIFT as a tool for studying attention in reading, we explored a number of different eye-movement measures reflecting different aspects of foveal and parafoveal processing that might potentially be affected by flickering. Firstly, we used the three classically reported fixation duration measures as dependent variables, considered to reflect different (earlier and later/more comprehensive) stages of foveal word processing and temporal planning of saccades (the “when” decisions):

1. First-fixation duration (FFD; duration in ms of the initial first-pass fixation on the word),
2. First-pass gaze duration (GD; duration in ms of the sum of all fixations on the word during the first-pass reading),
3. Total fixation duration (TFD; duration in ms of the sum of all fixations on the word, which includes any refixations).

Secondly, we used two eye-tracking measures which are more indicative of spatial planning of saccades (the “where” decisions):

(4) Incoming saccade landing position (SLP; difference in visual degrees between the *x*-coordinates of the first fixation on the target word and the centre of the word), and
(5) Incoming saccade amplitude (SA; distance in visual degrees from the start to the end point of a saccade initiated towards the target word).

Fixation and saccade events were extracted from the eye-tracker output files with custom MATLAB scripts. Preprocessing for the LME analyses involved exclusion of fixations shorter than 80 ms or more than 2.5 standard deviations longer than the mean per participant. After that, we log-transformed all fixation duration measures to avoid the rightward skew in the data distribution. We used “lmer()” function from *lmerTest* R package (Kuznetsova et al., 2017) to fit the models and “emmeans()” with “eff_size()” functions of the *emmeans* package for post hoc comparisons of interest and effect size calculation. The Holm correction for multiple comparisons was applied.

In each model, lexical frequency (high/low) and tagging condition (0/60/30 Hz) were treated as fixed factors. In addition, for fixation duration measures, we examined both the target and the pretarget words to capture any possible effects that tagging exerts on parafoveal processing. We used sum coding for factor levels with independent variables centred on the mean (following the recommendation by Brysbaert & Debeer, 2025) and fitted the modes with the maximal converging random effects structure. For most independent variables, the maximal random effects structure was used: random intercepts for participants and items, by-participant random slopes for tagging frequency condition, lexical frequency condition and region of interest (target or pretarget), as well as by-item random slopes for tagging frequency and the lexical frequency condition. Exceptions are GD and TRT, for which the full model did not converge. In these cases, random slopes explaining the smallest proportion of variance (<0.5%) were excluded until the model converged.

#### Bayesian linear mixed-effect (LME) analysis

Bayesian LME analysis (not preregistered) was performed using the *brms* R package (Version 2.23.0; Bürkner, 2017, 2021), using the same data, variables, and model structure as the frequentist LME analysis. Following Angele and colleagues (2025), we used weakly informative priors for all regression coefficients (Normal(0,10)) and the “brms()” defaults for all other parameters. We additionally performed a sensitivity analysis, re-fitting all the models with more narrow and informative priors: normal(0,1) for fixed effects and normal(5.3,5) for the intercept for all fixation duration measures, normal(0,0.1) for fixed effects and normal(3.5,3) for the intercept for SA, and normal(0,0.1) for fixed effects and normal(−0.3,1) for the intercept for SLP. Each model was run for 5,000 iterations (1,000 for warmup), across four chains. All models converged successfully (*R* hat ≤ 1.01). For each model, similarly to the frequentist LME analysis, we also ran post hoc comparisons of interest with the *emmeans* package, with Holm correction for multiple comparisons. We report the median (coefficient *b*) and the standard error of the posterior distribution, and the 95% credible interval (95% CrI5) of the estimate. We assumed evidence of an effect if the 95% CrI of its parameter estimate did not cross zero.

#### Divergent point analysis (DPA)

The DPA is a survival analysis approach that enables a precise estimate of the time when a specific empirical effect first discernibly impacts fixation duration distributions, as indexed by a specific significance or magnitude criterion (Reingold et al., 2012; Reingold & Sheridan, 2014, 2018; Sheridan et al., 2013; Sheridan & Reingold, 2012). This analysis allows comparing the survival curves for fixation durations between the two experimental conditions of interest (e.g., HF and LF), where each survival curve depicts the probability of the fixation duration exceeding each ms threshold. As a result, DPA identifies the earliest point of significant divergence between these curves, which signifies the earliest time at which the effect of interest may manifest. For instance, regarding the lexical frequency effect, DPA analysis showed that the onset of this foveal effect is very rapid (approximately 120 ms) and relies heavily on parafoveal preprocessing (Sheridan & Reichle, 2016).

In this study, we used DPA to obtain a quantitative estimation of the time course of the lexical frequency effect under the no-tagging, RIFT, and visible tagging conditions through comparing the survival curves for the first fixation duration between the HF and LF target words across these three tagging conditions. We used three complimentary DPA procedures—Classical DPA (preregistered), Confidence Interval DPA (not preregistered), and Ex-Gaussian DPA (not preregistered)—striving to get convergent evidence and, therefore, obtain more robust evidence for the lexical frequency effect onset differences (or lack thereof). DPA was carried out in MATLAB, following the methodology outlined by Reingold and Sheridan (2014, 2018) and adapting their scripts for the purposes of the current study. For all the DPA procedures, first fixation durations longer than 600 ms were discarded as outliers, following Reingold and colleagues (2012). First-fixation durations on the HF and LF target words were used as input for all the DPA procedures, and each of those procedures was applied separately to each of the three tagging conditions (no tagging, RIFT and visible tagging).

All the DPA procedures use a bootstrap resampling procedure with replacement (Efron & Tibshirani, 1994) to estimate the divergence point between the two survival curves, but use slightly different approaches to establish the divergence point. The Classical DPA procedure makes use of 10,000 iterations of random resampling in fixation durations for each participant and condition. During each iteration, for each ms bin, the survival probability for HF and LF words is calculated separately and averaged across participants. The difference between the survival probabilities for HF and LF words is then used to create a distribution for each ms bin, sorted in magnitude. Significance of the difference for a specific bin is established if the lower boundary of the confidence interval (the fifth lowest difference value) exceeds zero. The earliest ms bin in a row of five consecutive significant bins is considered as the divergence point and the onset of the lexical frequency effect.

Confidence Interval DPA is a modification of the Classical DPA, which appears to be more robust in cases of low experimental power and allows computing confidence intervals for the divergence point estimates to compare the onset of the divergence point across different conditions. It calculates a divergence point for each of 1,000 bootstrap iterations and uses a different criterion for the divergence point compared with the Classical DPA. Namely, this procedure uses a magnitude criterion (i.e., a difference between conditions of 1.5% or greater) for establishing the divergence point. The first ms bin in a run of five significant consecutive bins is then considered a divergence point for each iteration. The divergence point estimates obtained for each iteration are then sorted from the smallest to the largest value, with 25th and 975th values constituting the 95% confidence interval, while the median serves as the divergence point estimate for the sample. The results from this procedure are of special interest for the current study, since the confidence intervals it provides are expected to serve as indicators of a significant difference in divergence point estimates across the three different tagging conditions.

Ex-Gaussian DPA uses ex-Gaussian distribution fitting using quantile maximum likelihood estimation (QMPE; Cousineau et al., 2004; Heathcote et al., 2002). Ex-Gaussian parameters are obtained for each participant and the two lexical frequency conditions and are used to derive continuous probability density functions and survival curves. The first ms bin for which the survival percentage difference between high and low lexical frequency conditions constitutes at least 1.5% is considered a divergence point.

## Results

### Lexical frequency effect size: Linear mixed-effects analysis

**Fig. 3:**
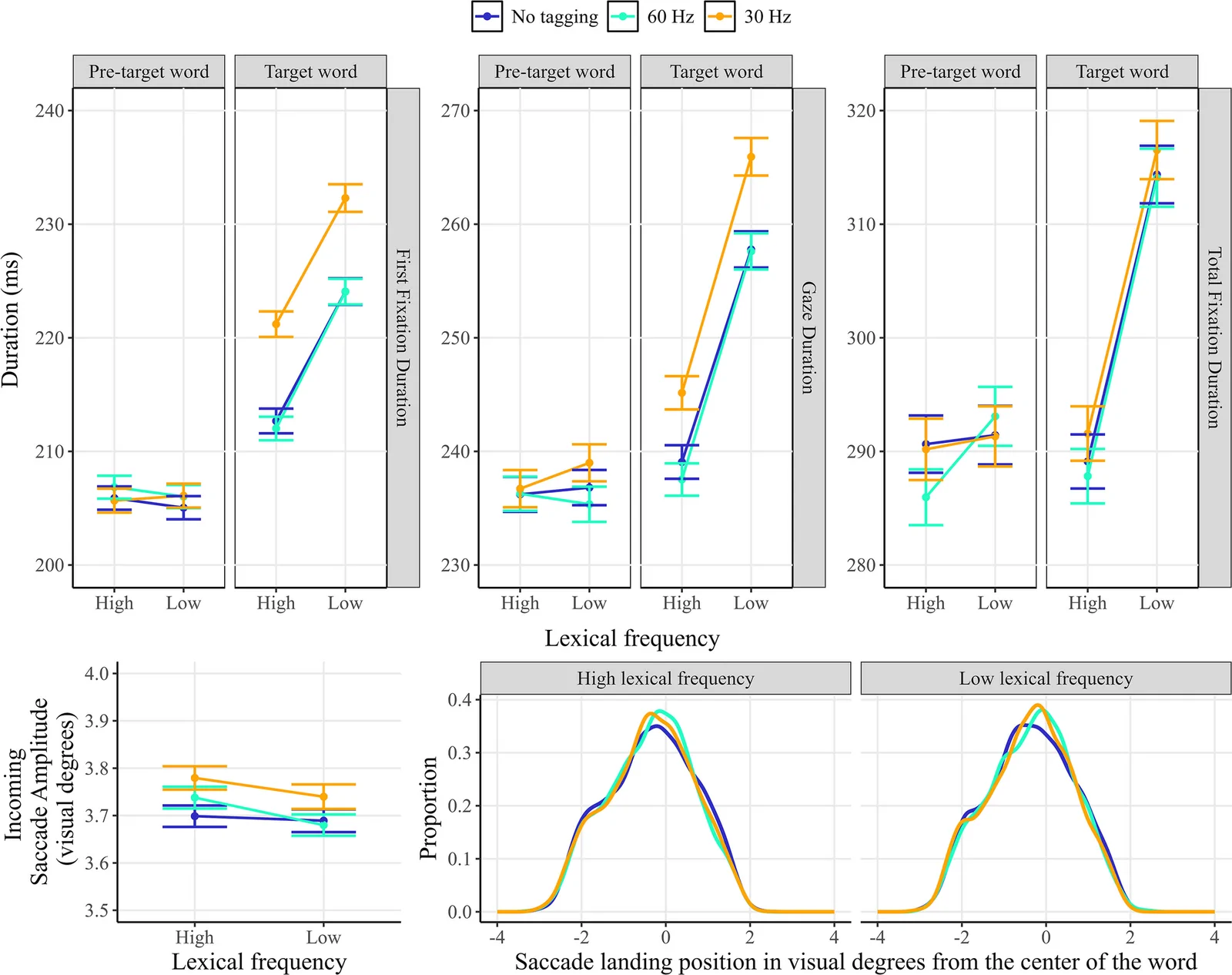
Fixation duration and incoming saccade amplitude means and standard errors and distributions of incoming saccade landing positions on the pretarget and target word depending on target word lexical frequency and tagging condition. Fixation duration data is presented for both pretarget and target word. Saccade amplitude and landing position data is presented for the target word only

Removal of fixation duration outliers (FFD: 3.16%; GD: 3.96%; TRT: 4.51%) resulted in retention of 4,028 observations per condition on average (*SD* = 143, range: 3,821–4,140) and 4,074 observations per condition on average (*SD* = 76.5, range: 3,962–4,164) for the target word. Two participants were lacking data for the last block (visible tagging in both cases), due to the experiment time constraints. Descriptive statistics can be found in Table 1 and Fig. 3:

**Table 1:** Descriptive statistics of eye movement measures for pretarget and target words

Tagging	No tagging		RIFT (60 Hz)		Visible tagging (30 Hz)	
Lexical frequency	Low	High	Low	High	Low	High
Pretarget words						
First fixation duration	205 (66)	206 (66)	206 (66)	207 (65)	206 (66)	206 (66)
Gaze duration	237 (99)	236 (98)	235 (98)	236 (96)	239 (101)	237 (100)
Total fixation duration	291 (164)	290 (159)	293 (164)	286 (156)	291 (162)	290 (165)
Target words						
First fixation duration	224 (76)	213 (69)	224 (73)	212 (66)	232 (77)	221 (71)
Gaze duration	258 (101)	239 (94)	258 (102)	237 (91)	266 (104)	245 (92)
Total fixation duration	313 (159)	289 (152)	313 (161)	288 (152)	315 (159)	291 (150)
Landing position	−0.37 (1.03)	−0.34 (1.05)	−0.36 (1.01)	−0.36 (1.02)	−0.39 (1.01)	−0.36 (1.03)
Saccade amplitude	3.69 (1.55)	3.7 (1.44)	3.68 (1.47)	3.74 (1.48)	3.74 (1.65)	3.78 (1.56)

Mean (standard deviation) calculated over participants (*n* = 48). All fixation durations are measured in ms. Incoming saccade landing position and amplitude are measured in visual degrees (°). For the incoming saccade landing position, the measure is relative to the centre of the word. Fixation duration data are presented for both pretarget and target word. Saccade amplitude and landing position data is presented for the target word only

We observed no *overall differences* in fixation duration on the pretarget word, but significant differences on the target word between the visible tagging and the other two tagging conditions (Table 2). FFD and GD were significantly longer for the visible target condition compared with both the RIFT and the no-tagging conditions, which did not differ from each other. This was found for both HF and LF target words. No overall differences were found in target word TFD or for the incoming saccade landing position and amplitude on the target word.

**Table 2:** Post hoc contrasts for the fixed effect estimates from the frequentist linear mixed-effects models

Factor	Pretarget word					Target word				
	*b*	*SE*	|*t*|	Sign.	d^a^_v_	*b*	*SE*	|*t*|	Sign.	d^a^_v_
First fixation duration										
**Intercept**	**5.307**	**0.024**	**223.189**	***						
**0 Hz: High − Low**	0.006	0.006	<1		0.023	**−0.05**	**0.006**	**8.319**	***	**−0.193**
**60 Hz: High − Low**	0.005	0.006	<1		0.018	**−0.051**	**0.006**	**8.539**	***	**−0.197**
**30 Hz: High − Low**	−0.001	0.006	<1		−0.003	**−0.044**	**0.006**	**7.263**	***	**−0.171**
High: 0 Hz − 60 Hz	−0.005	0.008	<1		−0.02	0.000	0.008	<1	−0.001	
Low: 0 Hz − 60 Hz	−0.006	0.008	<1		−0.024	−0.001	0.008	<1		−0.005
**High: 0 Hz − 30 Hz**	0.004	0.009	<1		0.017	**−0.043**	**0.009**	**4.925**	***	**−0.165**
**Low: 0 Hz − 30 Hz**	−0.002	0.009	<1		−0.009	**−0.037**	**0.009**	**4.280**	***	**−0.142**
**High: 60 Hz − 30 Hz**	0.009	0.009	1.001		0.036	**−0.043**	**0.009**	**4.543**	***	**−0.164**
**Low: 60 Hz − 30 Hz**	0.004	0.009	<1		0.015	**−0.036**	**0.009**	**3.819**	***	**−0.137**
Gaze duration										
**Intercept**	**5.413**	**0.029**	**187.947**	***						
**0 Hz: High − Low**	−0.001	0.007	<1		−0.003	**−0.076**	**0.007**	**−10.645**	***	**−0.243**
**60 Hz: High − Low**	0.005	0.007	<1		0.015	**−0.075**	**0.007**	**−10.503**	***	**−0.239**
**30 Hz: High − Low**	−0.010	0.007	−1.381		−0.033	**−0.076**	**0.007**	**−10.547**	***	**−0.243**
High: 0 Hz − 60 Hz	0.000	0.010	<1		−0.001	0.001	0.010	<1		0.003
Low: 0 Hz − 60 Hz	0.005	0.010	<1		0.017	0.003	0.010	<1		0.008
**High: 0 Hz − 30 Hz**	0.003	0.010	<1		0.010	**−0.034**	**0.010**	**−3.410**	**	−0.109
**Low: 0 Hz − 30 Hz**	−0.006	0.010	<1		−0.020	**−0.034**	**0.010**	**−3.427**	**	−0.109
**High: 60 Hz − 30 Hz**	0.003	0.011	<1		0.011	**−0.035**	**0.011**	**−3.319**	**	−0.112
**Low: 60 Hz − 30 Hz**	−0.012	0.011	−1.093		−0.037	**−0.037**	**0.011**	**−3.466**	**	−0.117
Total fixation duration										
**Intercept**	**5.562**	**0.036**	**153.794**	***						
**0 Hz: High − Low**	0.000	0.009	<1		0.001	**−0.088**	**0.009**	**−9.967**	***	**−0.222**
**60 Hz: High − Low**	−0.016	0.009	−1.800		−0.040	**−0.089**	**0.009**	**−10.054**	***	**−0.223**
**30 Hz: High − Low**	−0.009	0.009	<1		−0.023	**−0.085**	**0.009**	**−9.521**	***	**−0.214**
High: 0 Hz − 60 Hz	0.011	0.015	<1		0.028	0.002	0.015	<1		0.005
Low: 0 Hz − 60 Hz	−0.005	0.015	<1		−0.013	0.002	0.015	<1		0.004
High: 0 Hz − 30 Hz	0.001	0.014	<1		0.002	−0.028	0.014	−1.977		−0.070
Low: 0 Hz − 30 Hz	−0.009	0.014	<1		−0.022	−0.025	0.014	−1.768		−0.284
High: 60 Hz − 30 Hz	−0.011	0.016	<1		−0.026	−0.030	0.016	−1.885		−0.075
Low: 60 Hz − 30 Hz	−0.004	0.016	<1		−0.009	−0.026	0.016	−1.663		−0.066
Saccade landing position										
**Intercept**						**−0.355**	**0.038**	**−9.322**	***	
0 Hz: High − Low						0.029	0.022	1.333		0.030
60 Hz: High − Low						−0.003	0.022	<1		−0.003
30 Hz: High − Low						0.029	0.022	1.291		0.029
High: 0 Hz − 60 Hz						0.021	0.03	<1		0.021
Low: 0 Hz − 60 Hz						−0.012	0.03	<1		−0.012
High: 0 Hz − 30 Hz						0.014	0.028	<1		0.015
Low: 0 Hz − 30 Hz						0.014	0.027	<1		0.014
High: 60 Hz − 30 Hz						−0.006	0.028	<1		−0.006
Low: 60 Hz − 30 Hz						0.025	0.028	<1		0.026
Saccade amplitude										
**Intercept**						**3.767**	**0.102**	**37.022**	***	
0 Hz: High − Low						0.022	0.033	<1		0.016
60 Hz: High − Low						0.073	0.033	2.243		0.053
30 Hz: High − Low						0.051	0.033	1.528		0.037
High: 0 Hz − 60 Hz						−0.036	0.061	<1		−0.026
Low: 0 Hz − 60 Hz						0.015	0.061	<1		0.011
High: 0 Hz − 30 Hz						−0.076	0.063	−1.201		−0.055
Low: 0 Hz − 30 Hz						−0.047	0.063	<1		−0.034
High: 60 Hz − 30 Hz						−0.039	0.069	<1		−0.029
Low: 60 Hz − 30 Hz						−0.062	0.069	<1		−0.045

High = high lexical frequency target word; Low = low lexical frequency target word; 0 Hz = No tagging; 30 Hz = Visible tagging; 60 Hz = RIFT; *p <.05, **p <.01, ***p <.001.

We also found no significant differences in the *lexical frequency effect size* between the tagging conditions (Table 3). In all tagging conditions, there was a significant lexical frequency effect on the target word fixation duration, such that HF words were fixated for a shorter time than LF words (11–12 ms difference for FFD; 19–21 ms difference for GD; 24–25 ms difference for TFD; all *p* values <.001; see Table 2). No such effect was observed on the pretarget word fixation durations or on the target word incoming saccade amplitude or landing position in any tagging condition.

**Table 3:** Post hoc contrasts for the fixed effect estimates from the linear mixed-effect models: Size of the lexical frequency effect (high – low)

Factor	Pretarget word				Target word			
	*b*	*SE*	*|t|*	Sign.	*b*	*SE*	*|t|*	Sign.
First fixation duration								
0 Hz − 60 Hz	0.001	0.008	<1		0.001	0.008	<1	
0 Hz − 30 Hz	0.007	0.008	<1		−0.006	0.008	<1	
60 Hz – 30 Hz	0.005	0.008	<1		−0.007	0.008	<1	
Gaze duration								
0 Hz − 60 Hz	−0.006	0.010	<1		−0.001	0.010	<1	
0 Hz − 30 Hz	0.009	0.010	<1		0.000	0.010	<1	
60 Hz – 30 Hz	0.015	0.010	1.500		0.002	0.010	<1	
Total fixation duration								
0 Hz − 60 Hz	0.016	0.013	1.298		0.001	0.013	<1	
0 Hz − 30 Hz	0.009	0.013	<1		−0.003	0.013	<1	
60 Hz – 30 Hz	−0.007	0.013	<1		−0.004	0.013	<1	
Saccade landing position								
0 Hz − 60 Hz					0.032	0.030	1.069	
0 Hz − 30 Hz					0.001	0.031	<1	
60 Hz – 30 Hz					−0.032	0.030	1.043	
Saccade amplitude								
0 Hz − 60 Hz					−0.051	0.043	1.198	
0 Hz − 30 Hz					−0.029	0.043	<1	
60 Hz – 30 Hz					0.022	0.043	<1	

0 Hz = No tagging; 30 Hz = Visible tagging; 60 Hz = RIFT; *p <.05, **p <.01, ***p <.001

### Lexical frequency effect size: Bayesian linear mixed-effects analysis

Similarly to the frequentist analysis, there were no overall differences between RIFT and no-tagging condition, whereas for the 30-Hz tagging condition, FFD and GD on the target word were significantly longer than for the no-tagging and RIFT conditions, as suggested by the 95% CrIs below 0, for both high-frequency and low-frequency target words (Table 4). In addition, for all fixation duration measures on the target word there was a significant effect of lexical frequency, which effect size did not differ between the tagging conditions (Table 5). By contrast, for the pretarget word fixation durations (FFD, GD, TFD), as well as for the incoming saccade landing position on the target word, there were no significant effects of either lexical word frequency or tagging. Only for the incoming saccade amplitude and only in the RIFT condition there was a significant effect of target word lexical frequency, although its effect size did not differ significantly between the tagging conditions. Additional sensitivity analyses that used more informative priors yielded similar results (see Tables 9–10 in Supplementary Materials). To summarise, the Bayesian LME analysis provided evidence against any significant differences in the lexical frequency effect between the tagging conditions (except, possibly, for the saccade amplitude) and in favour of significantly longer overall target word fixations in the visible tagging condition, similarly to the LME analysis.

**Table 4:** Post hoc tests results from the Bayesian linear mixed-effects models on the eye-tracking measures

Contrast	Pretarget words				Target words			
	*b*	*SE*	95% CI		*b*	*SE*	95% CI	
			Lower	Upper			Lower	Upper
First fixation duration								
**Intercept**	**5.340**	**0.030**	**5.290**	**5.390**	**5.340**	**0.030**	**5.290**	**5.390**
**0 Hz: High − Low**	0.006	0.006	−0.006	0.018	**−0.050**	**0.030**	**−0.062**	**−0.039**
**60 Hz: High − Low**	0.005	0.006	−0.007	0.016	**−0.051**	**0.006**	**−0.063**	**−0.039**
**30 Hz: High − Low**	−0.001	0.006	−0.013	0.011	**−0.044**	**0.006**	**−0.057**	**−0.033**
High: 0 Hz − 60 Hz	−0.005	0.008	−0.021	0.011	0.000	0.006	−0.015	0.015
Low: 0 Hz − 60 Hz	−0.006	0.008	−0.022	0.009	−0.001	0.008	−0.016	0.015
**High: 0 Hz − 30 Hz**	0.004	0.009	−0.014	0.023	**−0.043**	**0.008**	**−0.061**	**−0.025**
**Low: 0 Hz − 30 Hz**	−0.002	0.009	−0.020	0.016	**−0.037**	**0.009**	**−0.055**	**−0.020**
**High: 60 Hz − 30 Hz**	0.009	0.010	−0.010	0.029	**−0.043**	**0.009**	**−0.062**	**−0.024**
**Low: 60 Hz − 30 Hz**	0.004	0.010	−0.015	0.023	**−0.036**	**0.010**	**−0.055**	**−0.016**
Gaze duration								
**Intercept**	**5.410**	**0.030**	**5.350**	**5.470**	**5.410**	**0.030**	**5.350**	**5.470**
**0 Hz: High − Low**	0.000	0.007	−0.015	0.014	**−0.075**	**0.008**	**−0.090**	**−0.061**
**60 Hz: High − Low**	0.005	0.007	−0.009	0.020	**−0.074**	**0.007**	**−0.088**	**−0.059**
**30 Hz: High − Low**	−0.009	0.008	−0.025	0.006	**−0.076**	**0.008**	**−0.091**	**−0.061**
High: 0 Hz − 60 Hz	0.000	0.010	−0.020	0.019	0.001	0.010	−0.019	0.021
Low: 0 Hz − 60 Hz	0.005	0.010	−0.014	0.025	0.002	0.010	−0.017	0.022
**High: 0 Hz − 30 Hz**	0.003	0.011	−0.018	0.024	**−0.034**	**0.011**	**−0.054**	**−0.013**
**Low: 0 Hz − 30 Hz**	−0.006	0.011	−0.027	0.015	**−0.034**	**0.011**	**−0.055**	**−0.013**
**High: 60 Hz − 30 Hz**	0.004	0.011	−0.018	0.026	**−0.035**	**0.011**	**−0.057**	**−0.013**
**Low: 60 Hz − 30 Hz**	−0.011	0.011	−0.033	0.011	**−0.036**	**0.011**	**−0.058**	**−0.014**
Total fixation duration								
**Intercept**	**5.560**	**0.040**	**5.490**	**5.640**	**5.560**	**0.040**	**5.490**	**5.640**
**0 Hz: High − Low**	0.000	0.010	−0.019	0.019	**−0.089**	**0.010**	**−0.108**	**−0.070**
**60 Hz: High − Low**	−0.017	0.010	−0.036	0.003	**−0.089**	**0.010**	**−0.108**	**−0.070**
**30 Hz: High − Low**	−0.010	0.010	−0.029	0.010	**−0.086**	**0.010**	**−0.106**	**−0.067**
High: 0 Hz − 60 Hz	0.011	0.016	−0.020	0.042	0.002	0.016	−0.030	0.032
Low: 0 Hz − 60 Hz	−0.006	0.016	−0.036	0.025	0.001	0.016	−0.029	0.032
High: 0 Hz − 30 Hz	0.001	0.015	−0.029	0.030	−0.027	0.015	−0.057	0.002
Low: 0 Hz − 30 Hz	−0.009	0.015	−0.038	0.020	−0.025	0.015	−0.055	0.004
High: 60 Hz − 30 Hz	−0.010	0.017	−0.043	0.024	−0.029	0.017	−0.062	0.005
Low: 60 Hz − 30 Hz	−0.003	0.017	−0.037	0.030	−0.026	0.017	−0.060	0.008
Incoming saccade landing position								
**Intercept**					**−0.340**	**0.040**	**−0.420**	**−0.260**
0 Hz: High − Low					0.029	0.022	−0.013	0.072
60 Hz: High − Low					−0.003	0.022	−0.046	0.039
30 Hz: High − Low					0.028	0.022	−0.015	0.071
High: 0 Hz − 60 Hz					0.022	0.030	−0.037	0.082
Low: 0 Hz − 60 Hz					−0.011	0.030	−0.069	0.048
High: 0 Hz − 30 Hz					0.015	0.029	−0.040	0.072
Low: 0 Hz − 30 Hz					0.014	0.029	−0.043	0.070
High: 60 Hz − 30 Hz					−0.007	0.030	−0.066	0.053
Low: 60 Hz − 30 Hz					0.024	0.029	−0.033	0.083
Incoming saccade amplitude								
**Intercept**					**3.800**	**0.120**	**3.570**	**4.040**
0 Hz: High − Low					0.022	0.033	−0.043	0.087
**60 Hz: High − Low**					**0.073**	**0.033**	**0.007**	**0.136**
30 Hz: High − Low					0.051	0.033	−0.015	0.116
High: 0 Hz − 60 Hz					−0.036	0.063	−0.158	0.088
Low: 0 Hz − 60 Hz					0.015	0.062	−0.107	0.137
High: 0 Hz − 30 Hz					−0.075	0.066	−0.203	0.057
Low: 0 Hz − 30 Hz					−0.046	0.066	−0.175	0.085
High: 60 Hz − 30 Hz					−0.039	0.072	−0.180	0.104
Low: 60 Hz − 30 Hz					−0.061	0.072	−0.202	0.079

High = high lexical frequency target word; Low = low lexical frequency target word; 0 Hz = No tagging; 30 Hz = Visible tagging; 60 Hz = RIFT.

**Table 5:** Differences in the size of the lexical frequency effect (high – low lexical frequency) between tagging conditions

	Pretarget word				Target word			
Contrast	*b*	*SE*	95% CrI		*b*	*SE*	95% CrI	
			Lower	Upper			Lower	Upper
First fixation duration								
0 Hz − 60 Hz	0.001	0.008	−0.015	0.018	0.001	0.008	−0.015	0.017
0 Hz − 30 Hz	0.007	0.008	−0.010	0.023	−0.006	0.008	−0.022	0.010
60 Hz − 30 Hz	0.005	0.008	−0.011	0.022	−0.007	0.008	−0.023	0.009
Gaze duration								
0 Hz − 60 Hz	−0.006	0.010	−0.025	0.013	−0.001	0.010	−0.020	0.018
0 Hz − 30 Hz	0.009	0.010	−0.010	0.029	0.000	0.010	−0.019	0.020
60 Hz − 30 Hz	0.015	0.010	−0.005	0.034	0.002	0.010	−0.018	0.021
Total reading time								
0 Hz − 60 Hz	0.017	0.012	−0.008	0.041	0.000	0.012	−0.024	0.024
0 Hz − 30 Hz	0.010	0.013	−0.015	0.035	−0.003	0.013	−0.028	0.022
60 Hz – 30 Hz	−0.007	0.013	−0.032	0.018	−0.003	0.013	−0.028	0.022
Incoming saccade amplitude								
0 Hz − 60 Hz					−0.051	0.043	−0.134	0.033
0 Hz − 30 Hz					−0.029	0.044	−0.114	0.057
60 Hz – 30 Hz					0.022	0.043	−0.063	0.107
Incoming saccade landing position								
0 Hz − 60 Hz					0.033	0.030	−0.026	0.091
0 Hz − 30 Hz					0.001	0.030	−0.057	0.063
60 Hz – 30 Hz					−0.032	0.030	−0.091	0.028

0 Hz = No tagging; 30 Hz = Visible tagging; 60 Hz = RIFT

### Lexical frequency effect onset: Divergence point analyses

**Fig. 4:**
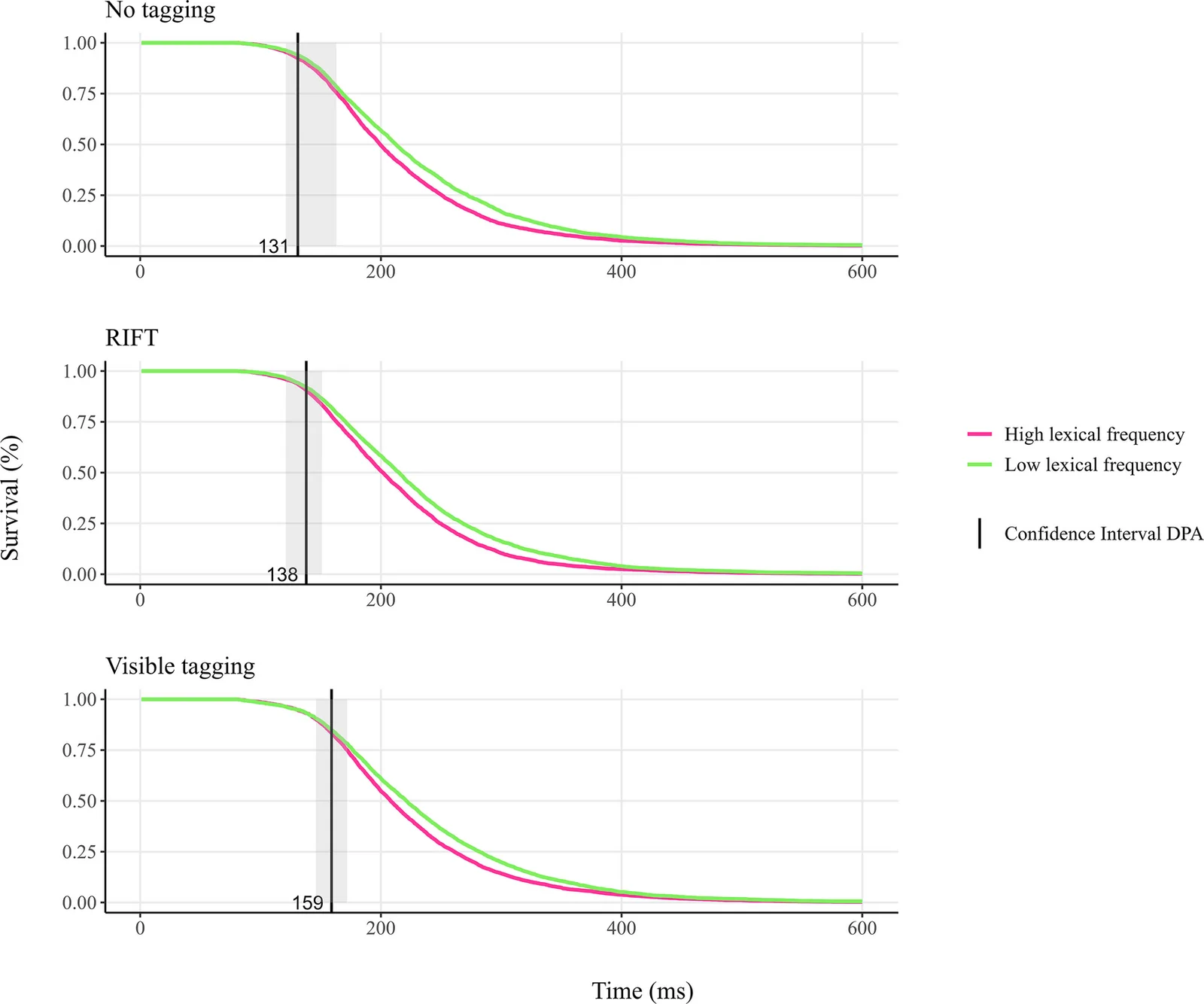
Confidence interval divergence point analysis results. Kaplan–Meier survival curves for the target word first fixation duration in high (pink line) and low (green line) lexical frequency conditions. Divergence point is represented by a vertical black line and its confidence interval with a light-grey rectangle area. Each plot represents results for a different tagging condition

Neither RIFT nor the visible tagging condition had significantly different divergence points compared to the no-tagging condition, as suggested by the Confidence Interval DPA procedure (Fig. 4). All of the DPA procedures provided similar divergence point estimates across each of the three tagging conditions, with very similar results for the no-tagging and RIFT conditions and higher, but not significantly higher, divergence point values for the visible tagging condition than for the other two conditions (Table 6). RIFT and the visible tagging conditions also had a 12–16-ms more narrow confidence interval than the no tagging condition (see Supplementary Materials for more details). Importantly, despite the slight numerical differences, confidence intervals overlapped to a great extent between all the tagging conditions, suggesting there are no significant differences in the lexical frequency effect onset.

**Table 6:** Divergence point analysis results

DPA procedure	No tagging	RIFT	Visible tagging
Classic DPA	137 ms	141 ms	166 ms
Confidence Interval DPA	131 [121, 163]	138 [121, 151]	159 [146, 172]
Ex-Gaussian DPA	142 ms	145 ms	167 ms

Median values in ms. For the confidence interval DPA, lower and upper boundary of the confidence interval are provided in square brackets

### Flickering awareness: *t*-test analysis

Due to a delayed introduction of the questionnaire to the experiment, we have data of 44 out of 48 participants for the flickering visibility question (whether there was flickering in a particular block) and data of 36 out of 48 participants for the perceived frequency. The data of eight participants, who misreported flickering in the no tagging condition, were excluded (18.18%), leaving us with 36 observations for the flickering visibility and 28 observations for the perceived frequency of the flickering.

There were significant differences between the three tagging conditions. While RIFT was reported to be noticed by 33% of participants overall (RIFT vs. no tagging), *t*(35) = 4.18, *p* <.001, it was significantly less perceptible than visible tagging (97%), *t*(35) = −7.87, *p* <.001. Furthermore, RIFT was more noticeable when it was in an experimental block following another tagging condition (55% after the no-tagging condition; 31% after the visible tagging condition) rather than when it was the first block in the experiment (17%). Although RIFT was present throughout the whole block, participants noticed it only in ~33% of the trials, as suggested by the mean flicker frequency rating on a 6-point Likert scale (*M* = 1.64, *SD* = 1.79; RIFT vs. no tagging), *t*(7) = 2.53, *p* =.04. Reported frequency of the visible flickering is much higher (~88%; *M* = 4.39, *SD* = 1.07; visible tagging vs. RIFT), *t*(17) = 4.85, *p* <.001.

## Discussion

Our goal was to examine whether RIFT and visible flickering of a word affected its lexical processing during natural reading. We considered the lexical frequency effect (e.g., Inhoff & Rayner, 1986; Rayner & Duffy, 1986; Schilling & Chumbley, 1998; Sheridan & Reichle, 2016; Staub et al., 2010; White, 2008) as a proxy and investigated whether RIFT (60 Hz) and visible tagging (30 Hz) affected its size and onset, as compared with reading without tagging. In line with previous studies (Inhoff & Rayner, 1986; Pan et al., 2021, 2023, 2025; Rayner & Duffy, 1986; Rayner et al., 1996; Schilling & Chumbley, 1998; Staub et al., 2010; White, 2008), high-frequency target words showed a processing advantage on all fixation duration measures for all tagging conditions during foveal fixation, whereas no lexical frequency effect was found during the fixation on the pretarget region or in landing position on the target word. Crucially, although a significant lexical frequency effect on incoming saccade amplitude was noted during RIFT modulation only, frequentist and Bayesian LME analyses, as well as DPA showed no significant differences in the magnitude or onset of the lexical frequency effect between the tagging conditions. At the same time, while there were no significant overall differences in reading patterns between RIFT and reading without tagging, first fixation and gaze durations were longer in the visible tagging condition as compared to RIFT and normal reading.

Our first question concerned the possible influence of RIFT on lexical word processing during reading. Several recent studies used this relatively novel method of studying covert attention to provide neural evidence in favour of parallel processing models of reading (Jensen et al., 2021; Pan et al., 2021, 2023, 2025), and consistently showed that individual reading speed is positively correlated with the use of the tagged parafoveal information. However, a reasonable objection to these findings would be that the flicker caused an increase in attentional resources being allocated to the tagged word, resulting in faster and/or more in-depth parafoveal processing. While some studies reported that invisible flickering does not affect spatial attention allocation (Alais et al., 2016), other studies showed adverse effects of high-frequency flickering on reading (Kennedy & Murray, 1991; Laycox et al., 2024; Le Floch & Ropars, 2023; Wilkins, 1986). In the present study, we found little evidence that foveal and parafoveal word processing in RIFT deviates from normal reading. The only difference was observed in incoming saccade amplitude: high-frequency words attracted a somewhat larger saccade than low-frequency words when they were flickered at an invisible frequency. This subtle effect is in line with saccade guidance phenomena in some previous studies showing that saccade amplitude can be guided by the upcoming word’s lexical frequency (Liu et al., 2015, 2016; Liu, Huang, Gao, et al., 2017a; Liu, Huang, Li et al., 2017b) and orthographic familiarity (Radach et al., 2004; but see White, 2008) and potentially reveals enhanced (sub-)lexical parafoveal processing of the invisibly tagged word. Notably, albeit significant, this effect is very small in magnitude (0.07˚), which constitutes less than a fifth of the space occupied by a single letter (0.5˚). Moreover, the meaningfulness of such an effect is questionable given that it is not observed in other related (e.g., target word landing position) and more robust measures (e.g., lexical frequency effect onset), as well as when comparing the size of the lexical frequency effect in different measures across tagging conditions.

Our second question was whether tagging visibility influences foveal and parafoveal word processing during reading. We hypothesized that visible tagging would either facilitate or disrupt lexical word processing to a greater extent than RIFT, due to consciously perceived visual attention manipulation. Despite that, we found no significant differences in either the magnitude or the onset of the lexical frequency effect, as compared to the no-tagging condition, suggesting that consciously perceived flickering is unlikely to affect lexical word processing. However, visible tagging did inflate overall first fixation and gaze durations on the tagged word, irrespective of the target word lexical frequency, suggesting more general interference of foveal visual processing of the tagged word. This effect is in line with previous studies, showing detrimental effect of low-frequency flickering on written word recognition in healthy adults (Lubineau et al., 2023) and its effectiveness as a spatial attention cue (Alais et al., 2016; Stolte & Ansorge, 2021).

Finally, we replicated previous findings on RIFT perceptibility. According to the postexperiment flickering awareness questionnaire, RIFT was consciously perceived by about 17% of the participants when it was the first experimental block,^6^ similarly to the usual RIFT detectability (Pan et al., 2021), and in only one third of the trials. When it followed another tagging condition, RIFT awareness increased to 31–55%. This supports the claim that RIFT manipulation proceeds mostly unnoticed when it is persistently used in the experiment, although there is some degree of individual variation.

To summarise, the current study holds significant practical value in guiding the future application of RIFT in natural reading research, as well as theoretical insights into the role of flickering awareness in reading behaviour. On the one hand, our study suggests that RIFT is unlikely to alter lexical word processing or attention allocation during reading, pointing at high ecological validity for this methodology. While employing frequencies higher than 60 Hz rarely results in a steady neural response in the visual cortex (Duecker et al., 2021), lower frequencies are more consciously perceived by the readers and seem to affect overall reading times. This makes RIFT (at 60 Hz) a valuable tool for measuring covert attention in reading, as it does not seem to alter written word processing, is largely unnoticeable, and ensures sufficient sensitivity to alternations in neural response.

As a consequence, current results also strengthen the validity of previous findings obtained using RIFT. Since no consistent effects of RIFT on parafoveal and foveal lexical processing were observed, it is relatively safe to assume that early lexico-semantic parafoveal-on-foveal effects (Pan et al., 2021, 2023), as well as temporally overlapping foveal and parafoveal effects in the tagging-induced signal (Pan et al., 2025), are unlikely to be a byproduct of flickering. Rather, they speak in favour of parallel lexical word processing models (Engbert et al., 2005; Jensen et al., 2021; Reilly & Radach, 2006; Snell, 2024; Snell et al., 2018) and alternative approaches allowing for partially parallel processing of several words (e.g., the Multi-Constituent Unit hypothesis (Zang, 2019) and the Oscillatory Pipelining Mechanism (Jensen et al., 2021).

On the other hand, the outcomes of this study suggest that low-frequency visible tagging (e.g., 30 Hz) actually reduces reading fluency. This supports the previous findings that different flicker frequencies have different impact on reading, both in healthy readers and readers with dyslexia (Le Floch & Ropars, 2023; Lubineau et al., 2023; McCloskey & Rapp, 2000; Pflugshaupt et al., 2007).

## Supplementary Information

Below is the link to the electronic supplementary material.

Supplementary file1 (DOCX 51 KB)

## Data Availability

The datasets generated during the current study and Supplementary Materials are available in the OSF repository (https://osf.io/bk52w/?view_only=5ea3d26f6c6847fbb2c20a34e09b50c4).

## Footnotes

1 Due to a mistake, the age and gender information is only available for the last 27 participants. The first half of the participants were recruited from the same pool of participants using the same inclusion criteria.

2 https://osf.io/65x27/?view_only=493b7e80f0e34b09993d78d53f140cbc

3 By bias we mean the decrease in accuracy and the increase in variability of divergence point estimates, simulated on the reduced subsets of data, either in terms of participants or trials (see Reingold & Sheridan, 2014, for more details).

4 Summary statistics for participants with and without MEG recordings can be found in the Supplementary Materials. In general, there are no major differences in the size of the lexical frequency effect between the groups, although participants with MEG recordings tend to have shorter fixation durations on both target and pretarget word.

5 See Supplementary Materials for more details about the RIFT setup.

6 This value is comparable with the change detection values for boundary change experiments (Slattery et al., 2011).

## Code availability

The codes generated during the current study are available in the OSF repository (https://osf.io/bk52w/?view_only=5ea3d26f6c6847fbb2c20a34e09b50c4).
