# Supplementary material for: Lexical word processing is unaffected by rapid invisible frequency tagging in reading: Evidence from eye movements
Source: Psychon Bull Rev. 2026 Jun 30;33(6):183. doi: 10.3758/s13423-026-02948-x (PMC13325337; doi:10.3758/s13423-026-02948-x)
Supplement: Supplementary file 1 — Supplementary file1 (DOCX 51 KB) [file 13423_2026_2948_MOESM1_ESM.docx]

**Stimuli sentence properties**

**Table 7**

*Properties of stimuli sentences and target words.*

| Dataset | N sentences/ target words | Freq. estim. | Lexical frequency | | Target word | | | | Sentence length |
| --- | --- | --- | --- | --- | --- | --- | --- | --- | --- |
|  |  |  | High | Low | Position | Length | Predict. | Plaus. |  |
| Pan et al. (2021), part of the stimuli | 78/78 | CELEX corpus | 95.3 (135.50) | 5.30 (4.50) | 6.70 (2.30) | 5.80 (0.80) | <0.10 | 5.60 (0.65) | 67.61 (3.72) |
| Degno et al. (2019) | 108/216 |  | Target word 1 | | | 5.59 (0.49) |  |  |  |
|  |  | English Lexicon Project | 291.96 (269.54) | 13.08 (5.89) | 3.74 (0.85) |  | <0.20 | 3.20–6.30 | 75.74 (4.23) |
|  |  |  | Target word 2 | | |  |  |  |  |
|  |  |  | 386.65 (650.96) | 12.39 (5.52) | 9.08 (1.18) |  |  |  |  |
| Schotter & Leinenger (2016) | 288/288 |  | 159.00 (208.00) | 4.00 (4.00) | 7.15 (1.20) | 5.88 (0.83) | 0.04 (0.07) | 5.35 | 62.61 (9.24) |

*Note.* Mean value (standard deviation). Lexical frequency is presented in items per million. Sentence length is presented in characters. Each study presented the results of the stimuli plausibility and predictability rating in a slightly different way: mean (standard deviation) in Pan et al. (2021), range in Degno et al. (2019), and just mean in Schotter & Leinenger (2016). Freq. estim. = Frequency estimates, Predict. = Predictability, Plaus. = Plausibility

**RIFT Procedure details**

To project stimuli at a sufficient refresh rate (1440 Hz), we displayed each sentence repeatedly in four quadrants of the stimulus computer screen (duplicated four times in each corner of the screen on each trial). Within each quadrant, the words were encoded in three colour channels as RGB. Consequently, the projector interpreted these 12 colour channels (3 channels × 4 quadrants) as 12 individual grayscale frames, which were then projected onto the projection screen in rapid succession. As a result, one frame from the stimulus computer screen was projected as 12 consecutive frames on the projection screen refreshed at 12 times the rate of the stimulus computer screen (120 Hz).

To prevent participants from noticing the edges of the patch during saccadic eye movements, a transparent mask was applied over the patch to smooth out its edges. This mask was generated using a two-dimensional smoothing Gaussian function:

$$mask = exp(-\frac{x^{2}+y^{2}}{{2\sigma}^{2}})$$

where, *x* and *y* are the mesh grid coordinates for the flickering patch, and σ is the x and y spread of the mask with σ = 0.02 degrees. The patch was perceived as middle-grey (RGB [128 128 128]), matching the colour of the background screen and effectively invisible to participants.

**Flickering awareness questionnaire**

1. Do you think there are some words flickering on the screen? In other words, the luminance of some words is changing all the time (yes/no question).
2. In which of the "big" blocks do you think there is flickering? (multiple-choice question: first, second, third)
3. If there was flickering in the **first** block, how often? (6-point Likert scale question: 0 – in 0% of the sentences
4. / never, 5 – in 100% of the sentences / always)
5. If there was flickering in the **second** block, how often? (6-point Likert scale question: 0 – in 0% of the sentences / never, 5 – in 100% of the sentences / always)
6. If there was flickering in the **third** block, how often? (6-point Likert scale question: 0 – in 0% of the sentences / never, 5 – in 100% of the sentences / always)

**Summary statistics for participants with and without MEG recordings**

**Table 8**

*Summary statistics for participants with and without MEG recordings.*

|  |  | | First fixation duration on the target word (ms) | | | | | | |  |
| --- | --- | --- | --- | --- | --- | --- | --- | --- | --- | --- |
|  | No tagging | | | | RIFT (60 Hz) | | | Visible tagging (30 Hz) | | |
| Participant group | HF | LF | | Diff. | HF | LF | Diff. | HF | LF | Diff. |
| Without MEG | 214 (69) | 226 (76) | | 12 | 217 (67) | 228 (74) | 11 | 228 (72) | 237 (78) | 9 |
| With MEG | 211 (69) | 223 (75) | | 12 | 208 (65) | 221 (72) | 13 | 215 (69) | 228 (76) | 13 |
|  |  | | First fixation duration on the pre-target word (ms) | | | | | | |  |
| Without MEG | 212 (68) | 209 (66) | | -3 | 213 (66) | 211 (66) | -2 | 213 (68) | 213 (67) | 0 |
| With MEG | 201 (63) | 202 (65) | | 1 | 202 (63) | 202 (65) | 0 | 199 (62) | 200 (64) | 1 |
|  |  | | Gaze duration on the target word (ms) | | | | | | |  |
| Without MEG | 245 (97) | 265 (106) | | 20 | 248 (97) | 267 (109) | 19 | 257 (96) | 277 (109) | 20 |
| With MEG | 234 (91) | 252 (98) | | 18 | 229 (86) | 250 (96) | 21 | 235 (88) | 256 (99) | 21 |
|  |  | | Gaze duration on the pre-target word (ms) | | | | | | |  |
| Without MEG | 247 (103) | 244 (102) | | -3 | 246 (99) | 245 (104) | -1 | 249 (105) | 251 (103) | 2 |
| With MEG | 228 (93) | 231 (96) | | 3 | 229 (93) | 227 (94) | -2 | 226 (95) | 229 (97) | 3 |
|  |  | | Total fixation duration on the target word (ms) | | | | | | |  |
| Without MEG | 303 (163) | 335 (180) | | 32 | 302 (156) | 326 (171) | 24 | 311 (158) | 336 (171) | 25 |
| With MEG | 278 (141) | 299 (145) | | 21 | 277 (148) | 305 (157) | 28 | 275 (142) | 300 (151) | 25 |
|  |  | | Total fixation duration on the pre-target word (ms) | | | | | | |  |
| Without MEG | 308 (170) | 303 (174) | | -5 | 296 (153) | 305 (168) | 9 | 313 (177) | 307 (166) | -6 |
| With MEG | 278 (151) | 282 (157) | | 4 | 278 (157) | 284 (163) | 6 | 270 (154) | 278 (159) | 8 |
|  |  | | Target word incoming saccade amplitude (˚) | | | | | | |  |
| Without MEG | 3.72 (1.54) | 3.64 (1.62) | | -0.08 | 3.69 (1.59) | 3.63 (1.47) | -0.06 | 3.85 (1.66) | 3.81 (1.77) | -0.04 |
| With MEG | 3.69 (1.36) | 3.73 (1.49) | | 0.04 | 3.77 (1.39) | 3.72 (1.46) | -0.05 | 3.72 (1.47) | 3.69 (1.54) | -0.03 |
|  |  | | Target word incoming saccade landing position (˚) | | | | | | |  |
| Without MEG | -0.28 (1.06) | -0.33 (1.05) | | -0.05 | -0.33 (1.03) | -0.33 (1.00) | 0 | -0.30 (1.04) | -0.31 (1.00) | -0.01 |
| With MEG | -0.39 (1.04) | -0.40 (1.01) | | -0.01 | -0.38 (1.01) | -0.37 (1.02) | 0.01 | -0.41 (1.02) | -0.46 (1.01) | -0.05 |

Note. *Mean (SD).* HF – High lexical frequency; LF – Low lexical frequency. Incoming saccade landing position is relative to the center of the target word.

**Bayesian Linear Mixed-effects analysis: sensitivity analysis with informative priors**

**Table 9**

*Post Hoc Tests Results from the Bayesian Linear Mixed-effects Models on the Eye-tracking Measures*

| First fixation duration | | | | | | | | | | | | |  |  |
| --- | --- | --- | --- | --- | --- | --- | --- | --- | --- | --- | --- | --- | --- | --- |
| Contrast | Pre-target words | | | | | | Target words | | | | | |  |  |
|  | *b* | | *SD* | 95% CI | | | *b* | | *SD* | 95% CI | | |  |  |
|  |  |  |  | Lower | | Upper |  |  |  | Lower | | Upper |  |  |
| **Intercept** | **5.27** | | **0.03** | **5.22** | | **5.32** | **5.27** | | **0.03** | **5.22** | | **5.32** |  |  |
| **0 Hz: High − Low** | 0.006 | | 0.006 | -0.006 | | 0.018 | **-0.050** | | **0.006** | **-0.062** | | **-0.039** |  |  |
| **60 Hz: High − Low** | 0.005 | | 0.006 | -0.007 | | 0.016 | **-0.051** | | **0.006** | **-0.063** | | **-0.040** |  |  |
| **30 Hz: High − Low** | -0.001 | | 0.006 | -0.013 | | 0.011 | **-0.044** | | **0.006** | **-0.056** | | **-0.032** |  |  |
| High: 0 Hz − 60 Hz | -0.005 | | 0.008 | -0.021 | | 0.011 | 0.000 | | 0.008 | -0.016 | | 0.016 |  |  |
| Low: 0 Hz − 60 Hz | -0.006 | | 0.008 | -0.022 | | 0.010 | -0.001 | | 0.008 | -0.017 | | 0.015 |  |  |
| **High: 0 Hz − 30 Hz** | 0.004 | | 0.009 | -0.014 | | 0.022 | **-0.043** | | **0.009** | **-0.061** | | **-0.025** |  |  |
| **Low: 0 Hz − 30 Hz** | -0.003 | | 0.009 | -0.021 | | 0.015 | **-0.037** | | **0.009** | **-0.055** | | **-0.019** |  |  |
| **High: 60 Hz − 30 Hz** | 0.009 | | 0.010 | -0.010 | | 0.029 | **-0.043** | | **0.010** | **-0.062** | | **-0.024** |  |  |
| **Low: 60 Hz − 30 Hz** | 0.004 | | 0.010 | -0.016 | | 0.023 | **-0.036** | | **0.010** | **-0.055** | | **-0.016** |  |  |
| Gaze duration | | | | | | | | | | | | |  |  |
| Contrast | Pre-target words | | | | | | Target words | | | | | |  |  |
|  | *b* | | *SD* | 95% CI | | | *b* | | *SD* | 95% CI | | |  |  |
|  |  |  |  | Lower | | Upper |  |  |  | Lower | | Upper |  |  |
| **Intercept** | **5.38** | | **0.03** | **5.32** | | **5.45** | **5.38** | | **0.03** | **5.32** | | **5.45** |  |  |
| **0 Hz: High − Low** | 0.000 | | 0.008 | -0.015 | | 0.014 | **-0.075** | | **0.008** | **-0.090** | | **-0.061** |  |  |
| **60 Hz: High − Low** | 0.005 | | 0.008 | -0.010 | | 0.020 | **-0.074** | | **0.007** | **-0.089** | | **-0.059** |  |  |
| **30 Hz: High − Low** | -0.010 | | 0.008 | -0.025 | | 0.006 | **-0.076** | | **0.008** | **-0.091** | | **-0.061** |  |  |
| High: 0 Hz − 60 Hz | 0.000 | | 0.010 | -0.020 | | 0.019 | 0.001 | | 0.010 | -0.019 | | 0.021 |  |  |
| Low: 0 Hz − 60 Hz | 0.005 | | 0.010 | -0.014 | | 0.025 | 0.002 | | 0.010 | -0.018 | | 0.022 |  |  |
| **High: 0 Hz − 30 Hz** | 0.003 | | 0.011 | -0.018 | | 0.024 | **-0.033** | | **0.010** | **-0.054** | | **-0.013** |  |  |
| **Low: 0 Hz − 30 Hz** | -0.006 | | 0.010 | -0.027 | | 0.015 | **-0.034** | | **0.010** | **-0.054** | | **-0.013** |  |  |
| **High: 60 Hz − 30 Hz** | 0.004 | | 0.011 | -0.018 | | 0.026 | **-0.034** | | **0.011** | **-0.056** | | **-0.012** |  |  |
| **Low: 60 Hz − 30 Hz** | -0.011 | | 0.011 | -0.033 | | 0.011 | **-0.036** | | **0.011** | **-0.058** | | **-0.015** |  |  |
| Total fixation duration | | | | | | | | | | | | |  |  |
| Contrast | Pre-target word | | | | | | Target word | | | | | |  |  |
|  | *b* | | *SD* | 95% CI | | | *b* | | *SD* | 95% CI | | |  |  |
|  |  |  |  | Lower | | Upper |  |  |  | Lower | | Upper |  |  |
| **Intercept** | **5.53** | | **0.04** | **5.45** | | **5.61** | **5.53** | | **0.04** | **5.45** | | **5.61** |  |  |
| **0 Hz: High − Low** | 0.000 | | 0.010 | -0.019 | | 0.019 | **-0.089** | | **0.010** | **-0.108** | | **-0.069** |  |  |
| **60 Hz: High − Low** | -0.016 | | 0.010 | -0.036 | | 0.003 | **-0.089** | | **0.010** | **-0.108** | | **-0.070** |  |  |
| **30 Hz: High − Low** | -0.009 | | 0.010 | -0.029 | | 0.011 | **-0.086** | | **0.010** | **-0.105** | | **-0.067** |  |  |
| High: 0 Hz − 60 Hz | 0.011 | | 0.016 | -0.020 | | 0.042 | 0.002 | | 0.016 | -0.029 | | 0.033 |  |  |
| Low: 0 Hz − 60 Hz | -0.005 | | 0.016 | -0.036 | | 0.026 | 0.002 | | 0.016 | -0.029 | | 0.033 |  |  |
| High: 0 Hz − 30 Hz | 0.001 | | 0.015 | -0.029 | | 0.031 | -0.027 | | 0.015 | -0.057 | | 0.003 |  |  |
| Low: 0 Hz − 30 Hz | -0.009 | | 0.015 | -0.038 | | 0.021 | -0.025 | | 0.015 | -0.054 | | 0.005 |  |  |
| High: 60 Hz − 30 Hz | -0.010 | | 0.017 | -0.044 | | 0.023 | -0.029 | | 0.017 | -0.063 | | 0.004 |  |  |
| Low: 60 Hz − 30 Hz | -0.003 | | 0.017 | -0.037 | | 0.030 | -0.026 | | 0.017 | -0.059 | | 0.007 |  |  |
| Incoming saccade landing position | | | | | | | | | | | | |  |  |
| Target word | | | | | | | | | | | | |  |  |
| Contrast | | *b* | | | *SD* | | | 95% CI | | | | |  |  |
|  |  |  |  |  |  |  |  | Lower | | | Upper | |  |  |
| **Intercept** | | **-0.350** | | | **0.040** | | | **-0.430** | | | **-0.270** | |  |  |
| 0 Hz: High − Low | | 0.024 | | | 0.020 | | | -0.015 | | | 0.062 | |  |  |
| 60 Hz: High − Low | | 0.002 | | | 0.020 | | | -0.037 | | | 0.041 | |  |  |
| 30 Hz: High − Low | | 0.027 | | | 0.021 | | | -0.014 | | | 0.068 | |  |  |
| High: 0 Hz − 60 Hz | | 0.012 | | | 0.025 | | | -0.036 | | | 0.060 | |  |  |
| Low: 0 Hz − 60 Hz | | -0.009 | | | 0.028 | | | -0.064 | | | 0.045 | |  |  |
| High: 0 Hz − 30 Hz | | 0.011 | | | 0.026 | | | -0.040 | | | 0.061 | |  |  |
| Low: 0 Hz − 30 Hz | | 0.014 | | | 0.028 | | | -0.041 | | | 0.068 | |  |  |
| High: 60 Hz − 30 Hz | | -0.001 | | | 0.027 | | | -0.053 | | | 0.051 | |  |  |
| Low: 60 Hz − 30 Hz | | 0.023 | | | 0.028 | | | -0.032 | | | 0.079 | |  |  |
| Incoming saccade amplitude | | | | | | | | | | | | |  |  |
| Contrast | *b* | | | *SD* | | | 95% CI | | | | | |  |  |
|  |  |  |  |  |  |  | Lower | | | Upper | | |  |  |
| **Intercept** | **3.77** | | | **0.11** | | | **3.55** | | | **3.99** | | |  |  |
| 0 Hz: High − Low | 0.026 | | | 0.028 | | | -0.029 | | | 0.081 | | |  |  |
| **60 Hz: High − Low** | **0.059** | | | **0.028** | | | **0.004** | | | **0.114** | | |  |  |
| 30 Hz: High − Low | 0.047 | | | 0.030 | | | -0.012 | | | 0.105 | | |  |  |
| High: 0 Hz − 60 Hz | -0.028 | | | 0.042 | | | -0.110 | | | 0.053 | | |  |  |
| Low: 0 Hz − 60 Hz | 0.005 | | | 0.041 | | | -0.077 | | | 0.084 | | |  |  |
| High: 0 Hz − 30 Hz | -0.038 | | | 0.052 | | | -0.139 | | | 0.063 | | |  |  |
| Low: 0 Hz − 30 Hz | -0.017 | | | 0.052 | | | -0.118 | | | 0.085 | | |  |  |
| High: 60 Hz − 30 Hz | -0.010 | | | 0.054 | | | -0.115 | | | 0.097 | | |  |  |
| Low: 60 Hz − 30 Hz | -0.022 | | | 0.054 | | | -0.127 | | | 0.084 | | |  |  |

**Table 10**

*Differences in the Size of the Lexical Frequency Effect (High – Low Lexical Frequency) between Tagging Conditions*

|  | Pre-target word | | | | Target word | | | |
| --- | --- | --- | --- | --- | --- | --- | --- | --- |
| Contrast | *b* | *SE* | 95% CI | | *b* | *SE* | 95% CI | |
|  |  |  | Lower | Upper |  |  | Lower | Upper |
| First fixation duration | | | | | | | | |
| 0 Hz − 60 Hz | 0.001 | 0.008 | -0.015 | 0.017 | 0.001 | 0.008 | -0.015 | 0.017 |
| 0 Hz − 30 Hz | 0.007 | 0.008 | -0.009 | 0.023 | -0.006 | 0.008 | -0.022 | 0.010 |
| 60 Hz − 30 Hz | 0.006 | 0.008 | -0.011 | 0.022 | -0.007 | 0.008 | -0.023 | 0.009 |
| Gaze duration | | | | | | | | |
| 0 Hz − 60 Hz | -0.006 | 0.010 | -0.025 | 0.013 | -0.001 | 0.010 | -0.020 | 0.018 |
| 0 Hz − 30 Hz | 0.009 | 0.010 | -0.010 | 0.029 | 0.000 | 0.010 | -0.019 | 0.019 |
| 60 Hz − 30 Hz | 0.015 | 0.010 | -0.005 | 0.034 | 0.002 | 0.010 | -0.017 | 0.021 |
| Total fixation time | | | | | | | | |
| 0 Hz − 60 Hz | 0.016 | 0.013 | -0.008 | 0.041 | 0.000 | 0.013 | -0.024 | 0.025 |
| 0 Hz − 30 Hz | 0.010 | 0.013 | -0.015 | 0.035 | -0.003 | 0.013 | -0.028 | 0.022 |
| 60 Hz – 30 Hz | -0.007 | 0.013 | -0.032 | 0.018 | -0.003 | 0.013 | -0.028 | 0.022 |
| Incoming saccade amplitude | | | | | | | | |
| 0 Hz − 60 Hz |  |  |  |  | -0.033 | 0.031 | -0.095 | 0.028 |
| 0 Hz − 30 Hz |  |  |  |  | -0.021 | 0.036 | -0.090 | 0.049 |
| 60 Hz – 30 Hz |  |  |  |  | 0.012 | 0.036 | -0.059 | 0.084 |
| Incoming saccade landing position | | | | | | | | |
| 0 Hz − 60 Hz |  |  |  |  | 0.021 | 0.025 | -0.027 | 0.069 |
| 0 Hz − 30 Hz |  |  |  |  | -0.003 | 0.027 | -0.057 | 0.050 |
| 60 Hz – 30 Hz |  |  |  |  | -0.024 | 0.027 | -0.077 | 0.029 |

*Note.* 0 Hz - No tagging; 30 Hz - Visible tagging; 60 Hz – RIFT

**Difference in Divergent Point Analysis (DPA) confidence intervals between the tagging conditions**

The RIFT and visible tagging conditions also had a 12-16 ms more narrow confidence interval than the no tagging condition. While future work is needed to further explore what may be driving the difference in the confidence intervals between the no tagging and the two frequency tagging conditions, it is most probably due to sample size differences between the tagging conditions. While there were no major differences in the overall number of observations between the three conditions (200-260 observations less for visible tagging due to lacking data), there are significant differences in sample size around the time of the divergence point (±25 ms around the divergence point). Indeed, the no tagging condition had fewer observations in that part of the distribution (n=1391) than the RIFT condition (n=1705) and even more so in comparison with the visible tagging (n=2096) condition. Thus, the number of observations around the divergence point might have had an impact on the CIs width. Alternatively, differences in CIs may also reflect sampling noise or decreased LFE onset variability during flickering, although none of the other eye-tracking variables seem to show less variability in the RIFT and visible tagging conditions.
